# Hybrid breeding of rice via genomic selection

**DOI:** 10.1111/pbi.13170

**Published:** 2019-06-26

**Authors:** Yanru Cui, Ruidong Li, Guangwei Li, Fan Zhang, Tiantian Zhu, Qifa Zhang, Jauhar Ali, Zhikang Li, Shizhong Xu

**Affiliations:** ^1^ Hebei Agricultural University Baoding China; ^2^ Department of Botany and Plant Sciences University of California Riverside CA USA; ^3^ National Key Laboratory of Crop Genetic Improvement and National Centre of Plant Gene Research (Wuhan) Huazhong Agricultural University Wuhan China; ^4^ Institute of Crop Science/National Key Facility for Crop Gene Resource and Genetic Improvement Chinese Academy of Agricultural Sciences Beijing China; ^5^ International Rice Research Institute Metro Manila Philippines; ^6^ Anhui Agricultural University Hefei China

**Keywords:** best linear unbiased prediction, cross‐validation, cytoplasm male sterile system, genomic prediction, hybrid rice

## Abstract

Hybrid breeding is the main strategy for improving productivity in many crops, especially in rice and maize. Genomic hybrid breeding is a technology that uses whole‐genome markers to predict future hybrids. Predicted superior hybrids are then field evaluated and released as new hybrid cultivars after their superior performances are confirmed. This will increase the opportunity of selecting true superior hybrids with minimum costs. Here, we used genomic best linear unbiased prediction to perform hybrid performance prediction using an existing rice population of 1495 hybrids. Replicated 10‐fold cross‐validations showed that the prediction abilities on ten agronomic traits ranged from 0.35 to 0.92. Using the 1495 rice hybrids as a training sample, we predicted six agronomic traits of 100 hybrids derived from half diallel crosses involving 21 parents that are different from the parents of the hybrids in the training sample. The prediction abilities were relatively high, varying from 0.54 (yield) to 0.92 (grain length). We concluded that the current population of 1495 hybrids can be used to predict hybrids from seemingly unrelated parents. Eventually, we used this training population to predict all potential hybrids of cytoplasm male sterile lines from 3000 rice varieties from the 3K Rice Genome Project. Using a breeding index combining 10 traits, we identified the top and bottom 200 predicted hybrids. SNP genotypes of the training population and parameters estimated from this training population are available for general uses and further validation in genomic hybrid prediction of all potential hybrids generated from all varieties of rice.

## Introduction

Hybrid breeding has been proven to be the most effective strategy to improve yield potential for many crops, especially in cross‐pollinated species such as maize, cotton and sorghum (Duvick, 2001; Khan *et al.*, 2009; Stephens and Holland, 1954). Hybrid breeding takes advantage of heterosis (hybrid vigour), which describes a phenomenon where F_1_ hybrids derived from crosses between genetically distinct inbred varieties exhibit superior phenotypic performances over their parents. Superior performances may be indicated by improved function of any biological process in the hybrid offspring (Stuber, 1994). The global food crisis has become a serious issue with the sharply increased human population in the world. In the next 20 years, the production of staple cereal grains, including corn, rice and wheat, must be doubled to keep up the pace with the world population growth (Zhao *et al.*, 2015a). Hybrid breeding is one of the effective ways to solve the world food crisis. Hybrid rice is a successful example of hybrid breeding, which has the potential of yielding ~20% more than the inbred varieties grown under the same conditions (Tu *et al.*, 2000). However, the process of developing rice hybrids is not simple, especially in terms of how to select parents for crosses (Xu *et al.*, 2014). It requires an extremely vast resource to evaluate field performances of all possible crosses among a large number of inbred lines (Schrag *et al.*, 2010). In general, only a small proportion of crosses can be evaluated in the field and many potential superior crosses may not have the opportunity to be tested. Marker assisted recurrent selection (MARS) is a vital method to identify novel genes and incorporate these genes into the recurrent parents for improvement of target traits (Bernardo, 2008; Collard and Mackill, 2008). Nevertheless, MARS has shown the weakness of a long selection cycle and inability to capture effects of minor genes. Furthermore, MARS is not the best practical method to develop hybrid crops by incorporating a few major genes or pyramiding several genes. In this context, genomic selection (GS) has paved the way to overcome these limitations using whole‐genome prediction models. GS is a form of MARS in which markers of the whole genome are used to predict the genomic values of individual lines or hybrids and has become an effective tool for plant breeding (Cooper *et al.*, 2014; Spindel *et al.*, 2015; Zhao *et al.*, 2015b). With the development of the next‐generation sequencing technology and the next‐generation phenotypic technology (high throughput phenotyping), GS can be a routine practice in plant breeding (Marulanda *et al.*, 2016).

Rice is a strictly self‐pollinated crop and commercial hybrid breeding depends on various types of male sterility systems. In a male sterility system, a male sterile line is incapable of setting seeds through selfing. Therefore, it can be used as the female parent to produce hybrids by receiving pollens from a normal fertile line. It has been a routine procedure to develop new hybrids through testcrosses between one or a few male sterile lines and many elite rice varieties or lines. The best hybrids can be identified based on their performances in experimental fields. The cost of phenotyping is typically high in hybrid breeding due to intensive labour and land use, and thus, development of hybrid rice is difficult. Genomic prediction of hybrid rice is promising because phenotypes of hybrids can be predicted before testcrosses are made and evaluated, which can significantly reduce the cost and labour. In genomic prediction of hybrid rice, models are developed from a training population where phenotypes are measured in the field and genotypes are deduced from their parents. New hybrids from the same set of parents form a test population whose phenotypes are predicted from the models learned from the training population.

Various prediction models for hybrid breeding have been developed, such as the best linear unbiased prediction (BLUP), the least absolute shrinkage selection operator (LASSO), Bayes B and empirical Bayes (Clark *et al.*, 2011; Habier *et al.*, 2013; Pasanen *et al.*, 2015). These methods often have similar performances in terms of prediction ability. However, when the number of markers and the sample size is very large, LASSO and other multiple regression‐based models often fail because these models cannot hold too many effects. The BLUP method is much more superior over the multiple regression‐based methods because markers are only used to infer the covariance structure of the mixed model and effects of these markers are not estimated (Wei and Xu, 2016; Xu *et al.*, 2014). Bernardo (1996a) applied the BLUP method to predicting single cross performances in maize and greatly enhanced the efficiency of commercial maize breeding programs. Prediction of hybrids has been investigated in rice breeding using BLUP with a marker inferred kinship matrix in place of pedigrees of the hybrids (Xu *et al.*, 2014). This proof‐of‐concept study showed that genomic hybrid breeding is very promising and offers an opportunity to solve the above‐mentioned problems in hybrid rice breeding.

**Figure 1:**
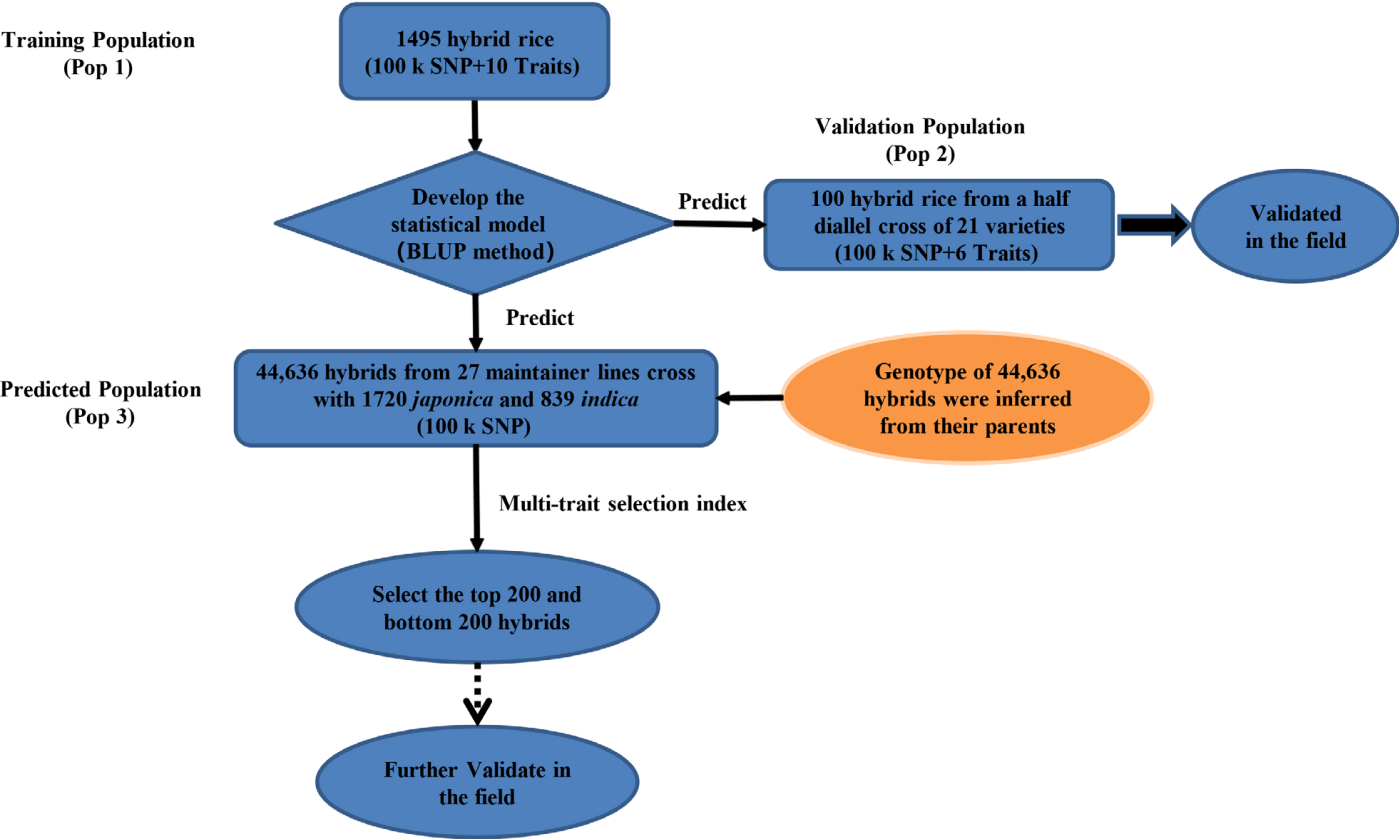
Flow chart of GS using the 1495 hybrid population as the training sample.

In addition to selection of the best models for genomic prediction, selecting training populations is another factor to consider. Hybrids in the training sample should have a diverse genetic background and should be derived from a wide range of different parents. Hybrids to be predicted should be related in some degree to the hybrids in the training sample. Rather than creating a new training sample of hybrids, which can be extremely costly, we can take advantage of the existing hybrids and use them as training populations for prediction. Huang *et al. *(2015) evaluated 1495 hybrids (Pop 1) in two field locations for a variety of agronomic traits for association studies and QTL mapping. These hybrids were derived from the crosses of a diverse panel of inbred lines. We took advantage of this hybrid population to perform hybrid prediction using replicated 10‐fold cross‐validations (CV). We then used one data set including 100 hybrids (Pop 2) from a half diallel cross experiment involving 21 parents to validate the prediction abilities of this hybrid population. The results showed good prediction for a number of agronomic traits. Finally, we predicted the performances of 44 636 potential hybrids (Pop 3) in a three‐line mating system obtained from the 3000 Rice Genome Project (Li *et al.*, 2014). Superior hybrids were predicted, and these hybrids are ready for field testing. If confirmed in the field by breeders, new hybrid cultivars could be developed relatively easily. A flow chart of GS using these three populations is presented in Figure 1.

## Result

### Prediction of a training population containing 1495 hybrids via cross‐validation

**Figure 2:**
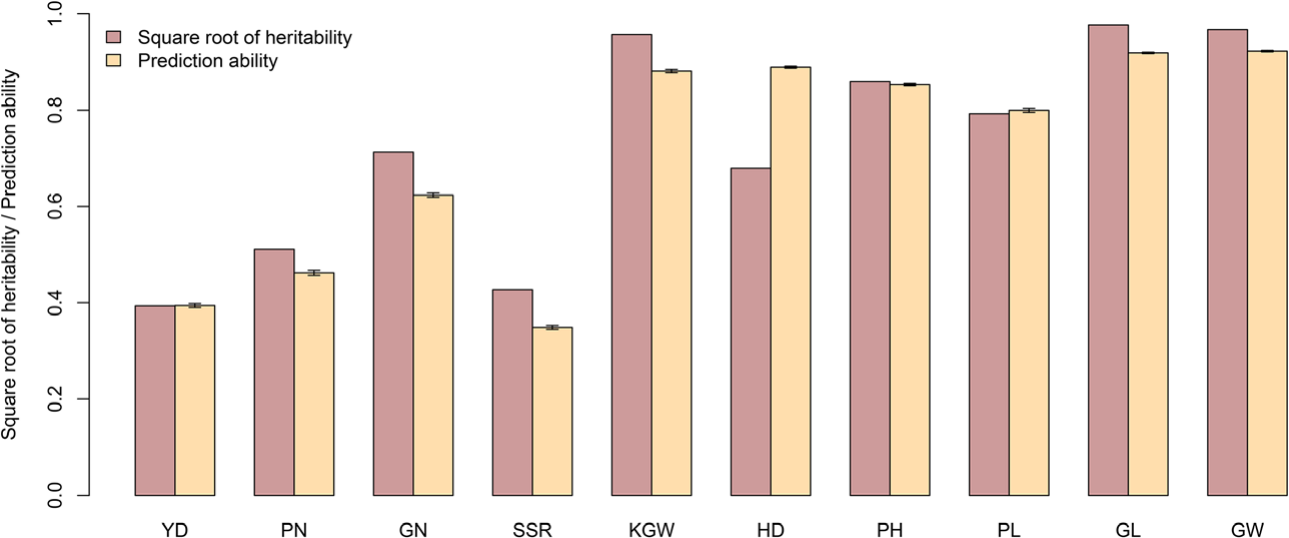
Estimated broad‐sense heritability and prediction ability for ten traits of the 1495 hybrid population. Heritability is defined as the genetic variance estimated from the ANOVA divided by the total phenotypic variance. Prediction ability is defined as the correlation between the predicted and observed trait values obtained from 10‐fold CV (replicated ten times).

The average prediction abilities drawn from replicated 10‐fold CV for ten agronomic traits are depicted in the bar‐plots of Figure 2 along with the square root of estimated heritability. Of the ten traits, seven have relatively high prediction abilities (greater than 0.6), including grain number (GN), 1000 grain weight (KGW), heading date (HD), plant height (PH), panicle length (PL), grain length (GL) and grain width (GW). We also noticed that the prediction ability is roughly proportional in the same direction to the square root of the estimated heritability. Figure S1 shows the plot of predicted phenotypes against the observed phenotypes for all ten traits. Since the prediction abilities of this hybrid population are reasonably high for all traits, we may use this training population to predict hybrids of other populations. To predict future hybrids, we need the estimated parameters from this hybrid population. These estimated parameters include the variance ratio $\hat{\lambda }$ and the population mean $\hat{\beta }$ for each trait. In addition, we also need the kinship matrix calculated from this hybrid population denoted by $K_{11}$. The prediction model also requires users to provide a covariance structure between the future hybrids and the hybrids in this training population denoted by $K_{21}$. The BLUP equation is given in Equation (13) and also presented here to improve the readability,

(1) $${\hat{y}}_{2}=E\left(y_{2}\left|y_{1}\right.\right)=X_{2}\hat{\beta }+\hat{\lambda }K_{21}{\left(K_{11}\hat{\lambda }+I\right)}^{-1}\left(y_{1}-X_{1}\hat{\beta }\right)$$

where ${\hat{y}}_{2}$ represents predicted trait values for future hybrids and *y*_1_ represents the trait values for the 1495 hybrids in this training population. The variance ratios ($\hat{\lambda }$) and the population means ($\hat{\beta }$) for all ten traits of the current hybrid population are given in Table S1. The kinship matrix *K*_11_ is given in Data S1. To calculate *K*_21_, users need to use their own SNP data that match the 102 795 SNPs of the current hybrid population.

### Independent validation from hybrids of a half diallel cross experiment

**Figure 3:**
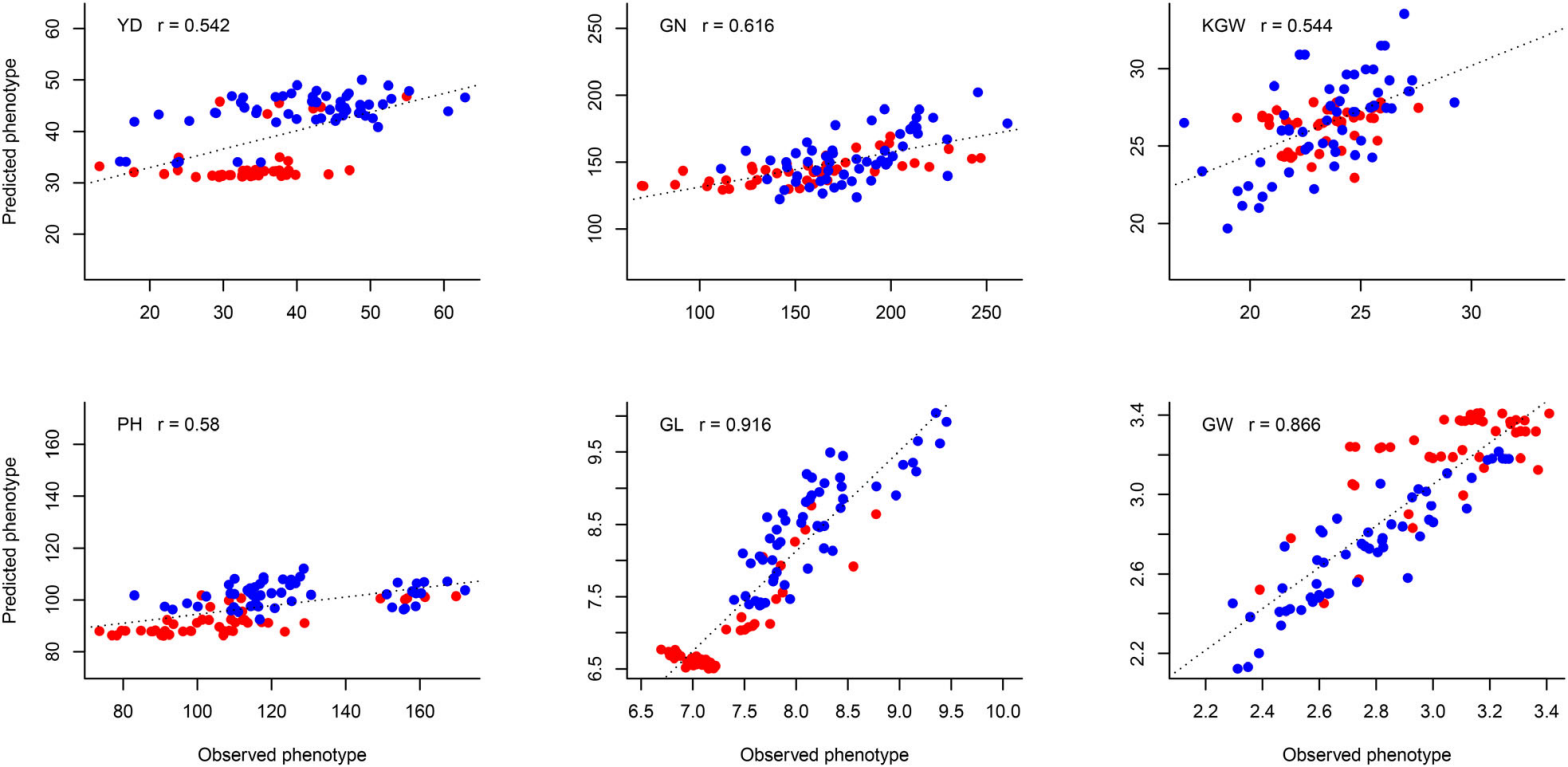
Plots of predicted phenotypes against observed phenotypes of 100 hybrid rice from the half diallel cross experiment. The training sample is the 1495 hybrid population. The six panels represent plots of six traits (YD, GN, KGW, PH, GL and GW) where *Geng* is colour coded in red and *Xian* is colour coded in blue.

We predicted 100 hybrids grown in Wuhan from a half diallel cross experiment using the pooled phenotype of 1495 hybrids of the training population for six traits. The prediction abilities for the six traits are 0.542, 0.616, 0.544, 0.580, 0.916 and 0.866, respectively, for YD, GN, KGW, PH, GL and GW. Plots of the predicted trait values against the observed trait values are shown in Figure 3. We also predicted the 100 hybrids using the phenotypic data in Sanya and Hangzhou, separately. The predicted phenotypes of the hybrids and the heritability are given in Data S2 and Table S2, respectively. The prediction abilities of the additive model using the phenotypic values collected from Hangzhou were higher than those using the phenotypic values measured from Sanya. The better prediction from Hangzhou was due to the fact that the photoperiod and temperature of Wuhan are similar to Hangzhou because they are located in the same latitude. However, the best prediction occurred when the pooled phenotypes of the two sites were used. The 21 parents of the diallel crosses, including 10 *Geng* (*japonica*) rice and 11 *Xian* (*indica*) rice, were not included in the parents of the 1495 hybrids; yet the prediction abilities were reasonably high for most of the traits. We concluded that the 1495 hybrid population may be used for prediction of hybrids derived from other inbred lines.

### Prediction of potential hybrids of 3000 germplasms

For 3000 germplasms, there are 3000 (3000 − 1)/2 = 4 498 500 potential hybrids. However, we only predicted 44 636 hybrids that are possibly grown in the field for commercial release because one parent of each hybrid of this kind is a maintainer line (see Materials and methods). The predicted phenotypes of these hybrids were given in Data S3 using the average phenotypic values of the training population collected in two locations (Hangzhou and Sanya). The phenotypic values of these potential hybrids predicted from the hybrids evaluated in Hangzhou and Sanya are given in Data S4 and S5, respectively. Information relevant to the 3000 germplasms is given in Data S6.

Although yield is the ultimate goal for improvement in hybrid breeding, it is not the only criterion to judge a hybrid. Other traits should also be considered in hybrid breeding. Therefore, we proposed a selection index to be considered as the criterion of hybrid breeding. The index is a linear combination of predicted values of all traits, each having a unique weight, as shown below,

(2) $$I_{j}=\sum_{k=1}^{10}w_{k}{\hat{y}}_{\mathrm{jk}}^{*}$$

where *I_j_* is the selection index score for individual *j*, *w_k_* is the economic weight for the *k*th trait for *k* = 1,2,…,10 (there are ten traits included in the index) and ${{\hat{y}}^{*}}_{\mathrm{jk}}$ is the standardized predicted value for trait *k* from the *j*th individual hybrid. To avoid duplicated usage of correlated traits, we divided nine traits (excluding yield) into three groups: the first group contained four yield components (PN, GN, SSR and KGW); the second group contained PL, GL and GW; and the last group contained HD and PH. Among the nine traits, the correlation coefficient between PL and PH is greater than 0.5 (Table S3), and this is the only trait pair with such a high correlation. All other correlations are below 0.5. Since the correlations are in general not high, we included all the ten traits in the selection index. The weights for the ten traits are

**Figure 4:**
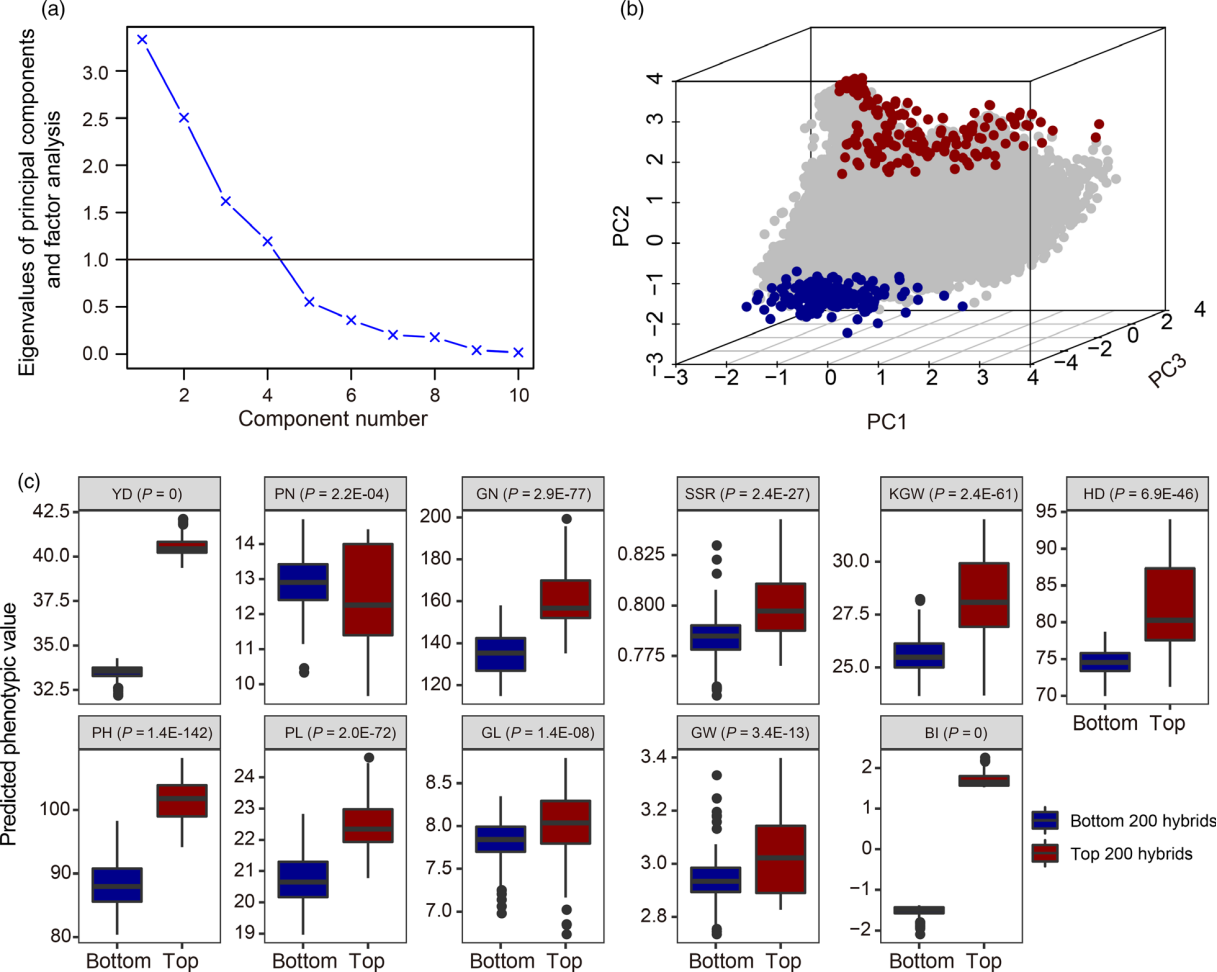
PCA plots and boxplots of selected hybrids. Panel (a): Eigenvalue plotted against the number of principal components for 10 predicted trait values from all 44 636 predicted hybrids (a 44 636 × 10 data matrix); Panel (b): 3D plot of the first three principal components of the 44 636 × 10 data matrix, where the best 200 and the worst 200 hybrids are colour coded blue and yellow, respectively; Panel (c): The differences between the top 200 hybrids and the bottom 200 hybrids for ten predicted traits and the BI.

(3) w=[0.40.10.10.10.050.050.050.050.050.05]

corresponding to the following order, YD, PN, GN, SSR, KGW, HD, PH, PL, GW and GL. Note that each weight is defined as a proportion. The weights were suggested by Zhao for rice breeding (Zhao *et al.*, 2015a). YD alone was weighted by 40%, all four yield components (PN, GN, SSR and KGW) accounted for 35%, the three grain quality traits (PN, GW and GL) were weighted by 15%, and the remaining two traits collectively took the remaining 10% (Zhao *et al.*, 2015a). For example, if the standardized predicted phenotypic value of YD of a particular hybrid is two, then the weight for YD should be 0.4 × 2 = 0.8. Adding the remaining nine traits the same way leads to an index value for a specific hybrid, we call this index value the breeding index (BI). Based on BI, we selected the top 200 and bottom 200 hybrids as candidates for evaluation in the future. We conducted a principal component analysis (PCA) for all the 44 636 predicted hybrids using the ten traits (predicted trait values) included in the index. The 3D plots of the first three principal components of the 44 636 × 10 data matrix showed clear separation of the two tails (Figure 4). We then presented boxplots for each trait in the selected top 200 and bottom 200 hybrids. The boxplots showed that all the ten traits and the BI were significantly different between the top and bottom predicted hybrids (Figure 4c).

### Dominance and G × E interaction for prediction of the 1495 hybrids

**Figure 5:**
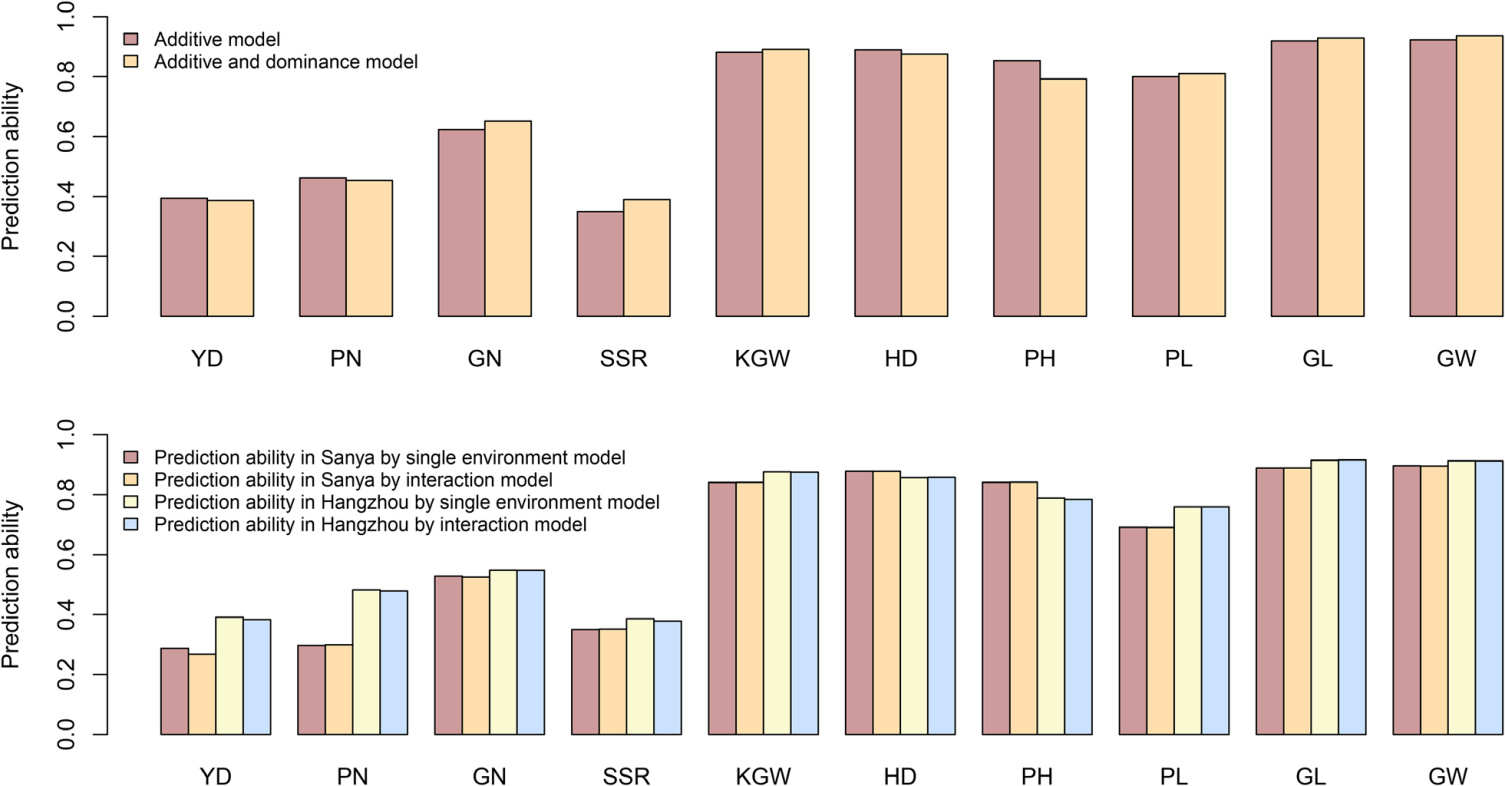
Prediction ability of different models. Panel (a): Prediction ability for ten traits of the rice population with 1495 hybrids under the additive and additive and dominance (A–D) models. Panel (b): Prediction ability of the G × E interaction model compared with environment specific prediction for ten agronomical traits.

We extended the additive model by incorporating dominance to form an additive and dominance (A‐D) model. We also extended the basic additive model to incorporate genotype by environment interaction (G × E) to form a G × E model. The A‐D and G × E models are described in Notes S1 and S2, respectively, where the G × E model uses a linear kernel (GBLUP) adopted from Cuevas et al. (Cuevas *et al.*, 2016). Figure 5a compares the prediction abilities of the additive model with the A‐D model for all ten traits of the hybrid population. Prediction abilities of the A‐D model were not significantly different from the additive model, that is we did not observe any expected improvement of the A‐D model over the additive model. Five of the ten traits showed slight improvement of the A‐D model, while the other five traits showed the opposite. The estimated variance components from the A‐D model are presented in Table S4. Figure 5b compares prediction abilities of the ten traits for the two locations separately and also jointly incorporating G × E interaction. To our surprise, the G × E model did not improve the prediction ability at all when compared with environment specific predictions. For some traits, for example YD, the G × E model actually decreased the prediction ability in both Hangzhou and Sanya, although the amount of decrease is barely noticeable.

## Discussion

### Prediction of potential hybrids of three‐line system from the 3000 germplasms

We used the 1495 hybrids of Huang *et al. *(2015) as the training population to predict potential hybrids of three‐line system of the 3000 lines from the 3K Rice Genome Project (Li *et al.*, 2014; Wang *et al.*, 2018). These potential hybrids may be directly applied to the three‐line hybrid breeding system depending on the availability of the fertility restoring genes in their male parents. We predicted the performance of a potential future population using the average phenotypes of hybrids in the training population evaluated in two environments. Results of prediction using the phenotypic values of individual environments separately were also conducted. The prediction ability using the Hangzhou phenotypic data was slightly higher than that using the Sanya phenotypic data, but neither one was better than that using the pooled phenotypic data (Figure S2). Based on the results of prediction from the pooled data, the selected top 200 and bottom 200 hybrids should be field evaluated in a future experiment. With the three‐line hybrid breeding system, we can only use inbred lines that have fertility restoring genes as the male parents to predict the hybrid progenies. Although this project is a pilot study attempting to predict potential hybrids of the three‐line breeding system in rice, the prediction model should be applied to hybrid breeding in all crops.

### Relationship between the training population and the test population

Conventional hybrid prediction requires that predicted hybrids must be related in pedigree to hybrids in the training sample (Massman *et al.*, 2013; Speed and Balding, 2015). When the pedigree relationships between the two sets of hybrids are unknown, Bernardo (1996b) proposed to use markers to estimate their relationships and then use BLUP to perform GS. This approach is later referred to as GBLUP by VanRaden (2008). The BLUP equation given in Equations (10) and (13) shows that a non‐zero *K*_21_ matrix is required. In the conventional BLUP, *K*_21_ = 0 if the two sets of hybrids are not genetically related. In genomic BLUP, however, *K*_21_ is essentially an identical by state matrix, which is never zero. This explains why GS using BLUP remains efficient for prediction of seemingly unrelated hybrids (de los Campos *et al.*, 2013). Wang *et al. *(2014) applied cross‐validation to calculate prediction ability under the situation where the predicted population is unrelated to the training population. In their study, the prediction ability was much higher when the training population was related to the test population than that when the two sets were not related. However, they only used 1048 diversity array technology markers in their analysis, and the training population size was very small. In our study, we used the 1495 hybrid training sample of Huang *et al *(2015) to predict 100 hybrids derived from an entirely different set but much more diverse parents and showed reasonable prediction ability. We treated the 1495 hybrids as a general training population to predict any future hybrids from any sources, especially the hybrids from *Xian‐Xian* crosses. It is widely recognized that the prediction ability of hybrids could be improved depending on the pedigree relationship between the training and test populations. However, using this generic training population of hybrids for genomic hybrid breeding is a very cost‐effective approach. Without this hybrid population, people would have to design a new cross experiment to generate a new training population with common parents shared by hybrids. Therefore, a general training population with diverse genetic backgrounds will be the key to predict hybrids in the future.

### Prediction ability and selection response

In the quantitative genetics theory, selection response is determined by heritability and selection intensity, as expressed in the breeders’ equation, *R* = *h* ^2^ *iσ*, where *h* ^2^ is the heritability, *i* is the selection intensity and *σ* is the phenotypic standard deviation of the trait under consideration. Thus, we can achieve a high response to selection for a low heritability trait with an extremely high selection intensity. The heritability of yield in our 1495 hybrid training population is about 0.16, and the square root of the heritability is $\sqrt{0.16}=0.4$. This root heritability should be compared with the prediction ability in GS. The prediction ability for yield does not seem to be high. However, we should be able to achieve a high gain by taking advantage of the high selection intensity. For example, the potential hybrids of the 3000 germplasms represent a hybrid population of 3000 (3000 − 1)/2 = 4 498 500. Selection of a few hybrids from this large pool means an extremely high selection intensity. Even though we only have a pool of 44 636 hybrids that are realistically selectable, they still represent a large base for GS. Therefore, genomic hybrid breeding is a very promising tool to improve grain yield in hybrid breeding of rice as well as other crops. Compared to GS using random populations where parameters have to be retrained in every few generations of selection to capture the changes of genetic parameters due to selection, genomic hybrid breeding is a one‐time event once a hybrid is identified, genomic hybrid breeding is done. Therefore, hybrid breeding is expected to benefit significantly from GS.

### Identification of fertility restoring genes

In this study, the top 200 predicted hybrids are derived from crosses between 17 maintainers and 61 other inbred lines. Of the 61 inbred lines, Minghui63 is the only elite rice restorer line that is known to carry a cloned restore gene complex *Rf1* on chromosome 10. Thus, to make use of the top 200 predicted hybrids in a three‐line hybrid breeding program, one must make sure all the 61 inbreds except for Minghui63 have restorer genes. In this respect, it is known that the *Rf1* complex consists of two genes named as *Rf1a* and *Rf1b*, while *Rf1b* is due to an amino acid alternation of Ser‐to‐Asn caused by a G‐to‐A variation responsible for fertility restoration (Akagi *et al.*, 2004). When examining the genomic sequences of the remaining 60 inbred lines, we also found that three lines contain the *Rf1b* gene. In addition to the *Rf1b* gene, there are 6 additional restore genes that have been cloned, including *Rf1a*, *Rf2*, *Rf4*, *Rf6*, *OsHXK6* and *GRP162* (Fujii *et al.*, 2014; Kazama and Toriyama, 2014; Wang *et al.*, 2006). Technically, molecular markers tightly linked to the six restore genes can be used as probes to test their existences in the rest of the inbred lines. If these restore genes are not carried by these lines, they can be easily imported by transgenic technology or by marker assisted through backcross breeding. With the available genome sequences and the pan‐genome data of the 3000 rice genomes (Sun *et al.*, 2017; Wang *et al.*, 2018), identification of the restorer genes in the parental lines of the top 200 hybrids is straightforward and no longer a limitation to apply our GS results to developing superior new rice hybrid cultivars of rice.

## Materials and methods

### Genetic material

#### Training population of hybrids

Three populations of rice were used in this project. The first one (Pop 1) is a training population that includes 1495 hybrids evaluated by Huang *et al. *(2015). Among the 1495 hybrids, 1361 hybrids were developed from the crosses between 288 male sterile lines and 621 restorer lines, and the rest have no pedigree information. Ten agronomic traits were investigated. They are yield (YD), PH, HD, panicle number per plant (PN), PL, grain number per panicle (GN), grain weight per 1000 grains (KGW), seed setting rate (SSR), GW and grain length (GL). The field experiments were conducted in the summer of 2012 in Hangzhou and in the winter of 2012 in Sanya. All the 1495 hybrids were grown on the farmland with well‐distributed soil status and uniform condition. Each hybrid was planted in a four‐row plot with six individuals in each row at a spacing of 20 cm × 2.6 cm in Hangzhou and 20 cm × 23 cm in Sanya. At maturity, three to five uniform plants in the middle of each plot were harvested to measure the ten agronomic traits. Of the 1495 hybrids, 1170 were generated from the three‐line mating system and the remaining 325 hybrids were bred from the two‐line mating system. The three‐line mating system contains cytoplasmic male sterility (CMS or A line), maintainer (B line) and restorer lines (R line). An A line is always multiplied by crossing with its B line. An R line possesses dominant fertility genes. When crossing to the A line, the R line restores fertility in the derived hybrids (see Figure S3). The two‐line mating system is also called environment sensitive genic male sterility. In this system, the male sterility condition depends on the interaction between the nuclear genes with an environmental factor such as photoperiod, temperature or both. The male sterility lines are multiplied by planting these lines in the environment that is conducive for inducing fertility. Hybrid seeds are produced by planting these lines in the environment that is conducive for inducing complete male sterility.

#### Validation population of hybrids from diallel crosses

The second test population (Pop 2) is a hybrid population including 210 hybrids which was derived from a half diallel cross experiment involving ten *Geng* rice and 11 *Xian* rice varieties (see Table S5). After removing 110 hybrids from the crosses between *Xian* and *Geng*, we only predicted 100 hybrids because these are within subspecies crosses that are practical in hybrid breeding. The field experiment was carried out in the summer growing seasons of 2013 on the Experimental Farm of Huazhong Agricultural University, Wuhan, China. The field planting followed a randomized complete block design with two replications (blocks). Each plot consisted of four rows with 10 plants per row, where the F_1_ hybrids were planted in the middle two rows and the two parents of the F_1_ hybrid were planted in the two peripheral rows. Seedlings of 30 days old were transplanted to the field, with a layout of 16.7 cm between plants within a row and 33.3 cm between rows. The field management followed the normal agricultural practice. Only eight plants in the middle of each row were used for phenotyping, and the plot mean was calculated and eventually used as the observed phenotypic value for the hybrid. Six traits were measured from the two replicated experiments. This population was used to test the prediction abilities for the training population of 1495 hybrids (the very first population). The six traits are YD, GN, KGW, PH, GL and GW. The average trait values of the two replicates (sites) were used as the phenotypic values for comparison with the predicted values. The prediction ability from this test population was defined as the correlation between the observed and the predicted phenotypic values for the trait of interest. The number of SNPs identified in the 21 parents was two million based on the version 7 map of *Geng* Nipponbare.

#### Potential hybrid population to be predicted

The third population (Pop 3) is the one released by the 3K Rice Genome Project (Alexandrov *et al.*, 2015). The 3000 germplasm accessions include 2466 accessions from the International Rice Gene Bank Collection at the International Rice Research Institute and 534 accessions from the China National Crop Gene Bank at the Institute of Crop Science, Chinese Academy of Agricultural Sciences. The 3000 rice accessions were collected from 89 countries and consist of two major groups *Xian* and *Geng*, a small group of intermediate varieties, and two small varietal groups aus/boro and aromatic basmati/sadri. Among the 3000 accessions, 27 varieties are maintainer lines. Of the remaining 3000 − 27 = 2973 lines, 58.2% are *Xian*, 27.9% are *Geng*, 9.4% are aromatic basmati/sadri and the last 4.5% are the intermediate type (see Figure S4). This population was used to generate potential hybrids for prediction.

Rice is a self‐pollinated crop, and there are no hybrids occurring in nature. To commercially develop hybrid rice, breeders rely on a three‐line mating system or a two‐line mating system. These systems depend on male sterile lines who can only accept pollens from other lines. There are no male sterile lines in the 3000 rice accessions. Since the nuclear genomes of the sterile lines and the maintainer lines are the same, we choose maintainer lines from the 3000 rice accessions as pseudomale sterile lines to infer the genotype of potential hybrids. Genotypes of the potential hybrids of the maintainer lines and conventional lines were deduced from the parental genotypes and then used to predict the phenotypic values. Of the 3000 accessions, 27 varieties are maintainer lines (23 *Xian* rice, three *Geng* rice and one intermediate type; see Table S6). Not all potential crosses of the maintainer lines with the conventional lines will be realistically developed in the field. Therefore, we did not predict the crosses of *Xian* × *Geng*; instead, we only predicted the crosses of *Xian × Xian*, *Geng × Geng*, intermediate type × *Xian* and intermediate type × *Geng*. There are a total of 23 × 1720 = 39 560 potential hybrids from *Xian × Xian*, 3 × 839 = 2517 potential hybrids from *Geng × Geng* and 1× (1720 + 839) = 2559 potential hybrids from intermediate type× (*Xian + Geng*), resulting in a total of 39 560 + 2517 + 2559 = 44 636 potential hybrids to be predicted.

Since no phenotypic values were collected from these potential hybrids, we were not able to test the prediction abilities of our models from this population. However, the top and bottom predicted hybrids in yield out of the 44 636 potential hybrids may be field evaluated in the future. If confirmed, the best top hybrids may be further tested in a large scale before released as new hybrid varieties. Field evaluation is beyond the scope of this study and will be deferred to a future project.

### SNP calling and genotype coding

The training population of 1495 hybrids was sequenced on Illumina Hiseq 2000 at twofold genome coverage, and 96‐bp paired‐end reads were generated. The raw sequence data set of the 1495 hybrids and the 1.6 million SNPs reference of the version 6 of *japonica* Nipponbare were released by Huang *et al. *(2015) in the rice haplotype project database. We downloaded the 2 TB raw sequence data of the 1495 hybrids and used version 7 of genomic pseudomolecule of *Geng* Nipponbare as the reference genome to call the SNPs. We did not use the reference of version 6 because sequences released by the 3000 Rice Genome Project were in version 7. This forced us to recall all SNPs from this updated version of reference. The version 7 genome of the *Geng* Nipponbare was downloaded from the website of Rice Genome Annotation Project (http://rice.plantbiology.msu.edu/annotation_pseudo_current.shtml). The downloaded paired‐end reads of all the hybrid rice accessions were aligned against the reference genome using the BWA software with the BWA‐MEM algorithm (Li and Durbin, 2009). For analysis of the 96‐bp reads, the BWA‐MEM algorithm is faster and more accurate than the other two algorithms within the BWA software package. The SNPs were identified using the SAMtools and BCFtools software packages. The alignments were done with a mapping quality ≥40, and the bases were called with a quality ≥20 when we identified the SNPs using SAMtools with the parameters ‐q20 ‐q40 of the subcommand mpileup. Overall, 1.6 million SNPs were called. The identified SNPs were further filtered by BCFtools. Only SNPs with the average depth (the overall depth divided by the number of varieties) for an individual being between 0.8 and 2.5 and the missing value of SNPs ≤25% were kept, leaving 232 935 SNPs in the data. Of the 232 935 SNPs, 201 756 overlap with SNPs of the 3000 germplasms whose potential hybrids were used for prediction.

There are 6.9 million SNPs for the 3000 germplasm accessions released by the 3K Rice Genome Project (Alexandrov *et al.*, 2015; Li and Durbin, 2009). These SNPs were called based on version 7 of the reference genome *japonica* Nipponbare. Among the 6.9 million SNPs, 201 756 of them overlap with the SNPs in the training population of 1495 hybrids (Table S7). These shared SNPs were converted into the BEAGLE (version 4.1) input file for imputation of missing SNPs. After screening for rare alleles with the MAF > 0.1 criterion, only 102 795 SNPs left in the data, which were used to generate potential hybrids of relevant varieties for GS.

The 102 795 common SNPs shared by both the training population and the predicted population were used to develop the prediction model and to predict phenotypes of potential hybrids in 3K rice genome programme. The genotypes of SNPs were coded by −1 for one homozygote, 0 for the heterozygote and 1 for the other homozygote. For example, if *A*_1_ is the reference allele and *A*_2_ is the alternative allele, there will be three possible genotypes, *A*_1_ *A*_1_, *A*_1_ *A*_2_ and *A*_2_ *A*_2_, whose numerical codes are −1, 0 and 1, respectively. Theoretically, all the 3000 germplasms should be homozygous at every locus and thus their potential hybrids should be coded the same way as we coded the genotypes for the 1495 hybrids in the training population. However, we occasionally encountered some heterozygotes in the 3000 germplasms, which led to uncertain genotypes of a potential hybrid. For example, when the mating type for the locus of interest was *A*_1_ *A*_1_ × *A*_1_ *A*_2_, the hybrid was then coded as (−1+0)/2 = −0.5. Similarly, when the mating type was *A*_2_ *A*_2_ × *A*_1_ *A*_2_, their hybrid was then coded as (0+1)/2 = 0.5. When the mating type was *A*_1_ *A*_2_ × *A*_1_ *A*_2_, their hybrid was coded as (−1+0+0+1)/4 = 0. In summary, the genotype of a potential hybrid can take the following numerical values: −1, −0.5, 0, 0.5 and 1, depending on the mating types of the parents.

### Statistical model for prediction

The BLUP method was used to perform prediction. The linear mixed model that includes all *m* markers is

(4) $$y=X\beta +\sum_{k=1}^{m}Z_{k}\gamma_{k}+\varepsilon =X\beta +\xi +\varepsilon$$

where *y* is an *n *× 1 vector of the phenotypic values of a quantitative trait measured from *n* individuals, *X* is a design matrix with rank *q* for all covariates, *β* is a vector of effects for the covariates (non‐genetic effects), $Z_{k}=\left\{Z_{\mathrm{jk}}\right\}$ is an *n* × 1 vector of genotype codes for marker *k* for individual *j*, *γ_k_* is the additive effect of marker *k* and *ε* is an *n* × 1 vector of residual errors with an assumed *N*(0,*Iσ* ^2^) distribution. The numerical code for individual *j* at locus *k* is defined as *Z_jk_* = −1 for *A*_1_ *A*_1_, *Z_jk_* = 0 for *A*_1_ *A*_2_, and *Z_jk_* = 1 for *A*_2_ *A*_2_. Assume that all marker effects follow a normal distribution with zero mean and a common variance, $\gamma_{k}\&x007E;N\left(0,\phi^{2}/m\right)$, for all *k* = 1,2,…,*m*, where *ϕ* ^2^ is an unknown polygenic variance. ξ is *n* × 1 vector of polygenic effect. When there is no other covariate except the intercept, *X* is an *n* × 1 vector of unity and *β* is simply a scalar of intercept. The expectation of *y* is *E*(*y*) = *X β* and the variance of *y* is var(*y*) = *V*, where

(5) $$V=\sum_{k=1}^{m}Z_{k}Z_{k}^{T}\phi^{2}/m+I\sigma^{2}=K\phi^{2}+I\sigma^{2}=\left(K\lambda +I\right)\sigma^{2}$$

with

(6) $$K=\frac{1}{m}\sum_{k=1}^{m}Z_{k}Z_{k}^{T}$$

being a marker inferred kinship matrix and $\lambda =\phi^{2}/\sigma^{2}$ is the variance ratio. The restricted log likelihood function for parameter λ is

(7) $$L\left(\lambda \right)=-\frac{1}{2}\ln|V|-\frac{1}{2}\ln\left|X^{T}V^{-1}X\right|-\frac{1}{2}{\left(y-X\beta \right)}^{T}V^{-1}\left(y-X\beta \right)$$

where

(8) $$\beta ={\left(X^{T}V^{-1}X\right)}^{-1}\left(X^{T}V^{-1}y\right)$$

and

(9) $$\sigma^{2}=\frac{1}{n-q}{\left(y-X\beta \right)}^{T}V^{-1}\left(y-X\beta \right).$$

After absorbing *β* and *σ* ^2^, the restricted log likelihood is only a function of *λ*. The Newton method of iteration was used to find the restricted maximum likelihood estimate of *λ*. To improve the computational speed, we also adopted the eigenvalue decomposition algorithm (Wei and Xu, 2016) for evaluation of the likelihood function.

Once parameters were estimated from the training population, they were used to predict the phenotypic values of the test population. Let *n*_1_ and *n*_2_ be the population sizes of the training and test populations, respectively. Let us also partition the phenotypic values into an *n*_1_ × 1 vector *y*_1_ for the training population and an *n*_1_ × 1 vector *y*_2_ for the test population. The polygenic effects and residual errors are also partitioned accordingly. The partitioned model is

(10) $$\left[\begin{array}{c} y_{1}\\ y_{2} \end{array}\right]=\left[\begin{array}{c} X\beta_{1}\\ X\beta_{2} \end{array}\right]+\left[\begin{array}{c} \xi_{1}\\ \xi_{2} \end{array}\right]+\left[\begin{array}{c} \varepsilon_{1}\\ \varepsilon_{2} \end{array}\right]$$

where

(11) $$E\left[\begin{array}{c} y_{1}\\ y_{2} \end{array}\right]=\left[\begin{array}{c} X\beta_{1}\\ X\beta_{2} \end{array}\right]$$

and

(12) $$\text{var}\left[\begin{array}{c} y_{1}\\ y_{2} \end{array}\right]=\left[\begin{array}{c} K_{11}\;K_{12}\\ K_{21}\;K_{22} \end{array}\right]\phi^{2}+\left[\begin{array}{c} I\;0\\ 0\;I \end{array}\right]\sigma^{2}.$$

Note that the *K* matrix has been partitioned into 2 × 2 blocks with *K*_11_ being the kinship matrix for the training sample, *K*_22_ being the kinship matrix for the test population and $K_{21}=K_{12}^{T}$ being the kinship between hybrids in the test population and hybrids in the training population. The BLUP of hybrids in the test population is expressed as

(13) $${\hat{y}}_{2}=E\left(y_{2}\left|y_{1}\right.\right)=X_{2}\hat{\beta }+\hat{\lambda }K_{21}{\left(K_{11}\hat{\lambda }+I\right)}^{-1}\left(y_{1}-X_{1}\hat{\beta }\right).$$

If *y*_2_ are observed, we can then calculate the prediction ability as the correlation coefficient between *y*_2_ and ${\hat{y}}_{2}$ as expressed below

(14) $$r_{y_{2}{\hat{y}}_{2}}=\frac{\mathrm{cov}(y_{2},{\hat{y}}_{2})}{\sqrt{\mathrm{var}\left(y_{2}\right)\mathrm{var}\left({\hat{y}}_{2}\right)}}.$$

We proposed to use the 1495 hybrids of the training population to predict 44 636 potential hybrids from 3000 rice accessions of the 3K genome project. In this case, *y*_1_ represents the phenotypic values of the 1495 hybrids, *y*_2_ represents the phenotypic values of the 44 636 hybrids, *n*_1_ = 1495, *n*_2_ = 44 636, *K*_11_ is a 1495 × 1495 matrix, *K*_22_ is a 44 636 × 44 636 matrix and *K*_21_ is a 44 636 × 1495 matrix.

We also considered a model incorporating dominance effects, called the additive and dominance (A‐D) model (Vitezica *et al.*, 2013). Details of the A‐D model are provided in Supplementary Note S1. The two locations (Hangzhou and Sanya) allowed us to investigate a G × E interaction model (Cuevas *et al.*, 2016). Therefore, we performed prediction separately for each environment and also jointly by incorporating the G × E interaction effects using the GBLUP model adopted from Cuevas *et al. *(2016). Details of the G × E model are provided in Note S2.

### K‐fold cross‐validation

Prediction ability of a method for a particular trait can be obtained from cross‐validation (Kohavi, 1995). The most commonly used cross‐validation is the K‐fold cross‐validation where the sample is partitioned into k roughly equal sized parts. Each part is predicted using parameters estimated from the other *K* − 1 parts (sample excluding the part to be predicted). Eventually, all parts are predicted using samples that exclude the parts to be predicted. We often choose *K* = 10 and thus call the method a 10‐fold cross‐validation (Figure S5). In a 10‐fold cross‐validation for the 1495 hybrids, each part contained roughly 150 hybrids. Since the prediction ability drawn from the 10‐fold cross‐validation depended on how the ten parts are partitioned, we replicated the cross‐validation experiment ten times and each time the sample was partitioned differently. The average prediction ability for each trait was reported. The prediction ability was calculated as the Pearson correlation coefficient between the observed and predicted phenotypic values.

### Heritability estimation from a completely randomized block design of experiment

Broad‐sense heritability, *albeit* not used in genomic prediction, was estimated for 10 traits of the 1495 hybrid population using the conventional method of analysis of variance under a completely randomized block design (CRBD) with the two locations treated as two blocks. The linear model for the CRBD design is

(15) $$y_{\mathrm{jk}}=\mu +\alpha_{j}+\beta_{k}+\varepsilon_{\mathrm{jk}}$$

where *y_jk_* is the phenotypic value of the trait of interest of the *j*th hybrid measured from the *k*th location for *j* = 1,2,…,*n*, *k* = 1,2 and n = 1495, *µ* is the grand mean, *α_j_* is the genetic effect of the *j*th hybrid, *β_k_* is the effect of the *k*th block and *ε_jk_* is the residual error (may contain a G × E interaction effect) with an assumed $N(0,\sigma_{E}^{2})$ distribution. This is a mixed model with *β_k_* treated as a fixed effect and *α_j_* as a random effect following a $N(0,\sigma_{G}^{2})$ distribution for all *j* = 1,2,…,*n*. The broad‐sense heritability is defined as

(16) $$h_{\text{broad}}^{2}=\frac{\sigma_{G}^{2}}{\sigma_{G}^{2}+\sigma_{E}^{2}}.$$

The two variance components were estimated from variance component analysis presented in Table S8 using the VARCOMP procedure in SAS (SAS Institute, 2009).

## Conflict of interest

The authors declare no conflict of interest.

## Authors’ contributions

Y. Cui and S. Xu designed the project and developed the statistical models for prediction. R. Li performed the SNP calling of 1495 hybrids. G. Li and Q. Zhang conducted the field trail and phenotyping. F. Zhang and T. Zhu participated in the development of statistical models. J. Ali and Z. Li provided the information of selection index. Y. Cui prepared the first draft and Z. Li and S. Xu revised the manuscript. All the authors approved the final version of the manuscript.

## Supporting information


**Data S1** The kinship matrix of K_11_.

**Data S2 **Predicted values of the 100 hybrids from the half diallel crosses.

**Data S3** Predicted values of potential hybrids from the 3000 germplasms for 10 agronomic traits (predicted from the average phenotypic values of the 1495 hybrids across two replicates).

**Data S4** Predicted values of potential hybrids from the 3000 germplasms for 10 agronomic traits (predicted from phenotypic values of the 1495 hybrids in Hangzhou).

**Data S5** Predicted values of potential hybrids from the 3000 germplasms for 10 agronomic traits (predicted from phenotypic values of the 1495 hybrids in Sanya).

**Data S6** Relevant information of 3,000 rice varieties of the 3K rice genome project.

**Note S1 **Additive & dominant (A‐D) model.
**Note S2 **G×E interaction model.
**Table S1** Estimated parameters for 10 traits of the 1495 hybrids.
**Table S2** Prediction abilities of the test population (Pop 2) using phenotypes collected from different locations of the training population (Pop 1).
**Table S3** Correlation matrix between 10 agronomic traits.
**Table S4** Estimated parameters for 10 traits of the 1495 hybrids from the additive‐dominant (A‐D) model.
**Table S5** Information of 21 inbred lines of the half diallel cross experiment (Pop 2).
**Table S6** Information of 27 maintainer lines of the 3K rice genome project (Pop 3).
**Table S7** Distribution of SNPs on the 12 chromosomes of rice.
**Table S8** ANOVA table for estimating variance components that are required for estimating the broad sense heritability.
**Figure S1** Predicted phenotypes plotted against observed phenotypes of the 1495 hybrid rice for 10 traits (YD, PN, GN, SSR, KGW, HD, PH, PL, GL and GW).
**Figure S2** Prediction abilities using phenotypes collected from Hangzhou and Sanya separately and prediction abilities using mean phenotype of the two sites (pooled).
**Figure S3** Three‐line mating system. The three‐line mating system involves three lines.
**Figure S4** Distribution of the 3,000 rice accessions from five distinct groups of the 3K Rice Genome Project.
**Figure S5 **Sketch of a K‐fold cross validation where K = 10.

## Data Availability

The DNA sequencing data referenced in this study are deposited in the European Nucleotide Archive (http://www.ebi.ac.uk/ena/) under accession number ERP005527. The 3k SNP data referenced in this study are available in the Rice SNP‐Seek Database (http://snp-seek.irri.org/). All other relevant data and R code are available from the corresponding authors upon request.

## References

[pbi13170-bib-0001] Akagi, H. , Nakamura, A. , Yokozeki‐Misono, Y. , Inagaki, A. , Takahashi, H. , Mori, K. and Fujimura, T. (2004) Positional cloning of the rice Rf‐1 gene, a restorer of BT‐type cytoplasmic male sterility that encodes a mitochondria‐targeting PPR protein. Theor. Appl. Genet. 108, 1449–1457. 14968308 10.1007/s00122-004-1591-2

[pbi13170-bib-0002] Alexandrov, N. , Tai, S. , Wang, W. , Mansueto, L. , Palis, K. , Fuentes, R.R. , Ulat, V.J. , et al. (2015) SNP‐Seek database of SNPs derived from 3000 rice genomes. Nucleic Acids Res. 43, 1023–1027. 10.1093/nar/gku1039 PMC4383887 25429973

[pbi13170-bib-0003] Bernardo, R. (1996a) Best linear unbiased prediction of maize single‐cross performance. Crop Sci. 36, 50–56. 10.1007/BF00230131 24162487

[pbi13170-bib-0004] Bernardo, R. (1996b) Testcross additive and dominance effects in best linear unbiased prediction of maize single‐cross performance. Theor. Appl. Genet. 93, 1098–1102. 24162487 10.1007/BF00230131

[pbi13170-bib-0005] Bernardo, R. (2008) Molecular markers and selection for complex traits in plants: learning from the last 20 years. Crop Sci. 48, 1649–1664.

[pbi13170-bib-0006] Clark, S.A. , Hickey, J.M. and Van der Werf, J.H. (2011) Different models of genetic variation and their effect on genomic evaluation. Genet. Sel. Evol. 43, 18. 21575265 10.1186/1297-9686-43-18 PMC3114710

[pbi13170-bib-0007] Collard, B.C. and Mackill, D.J. (2008) Marker‐assisted selection: an approach for precision plant breeding in the twenty‐first century. Philos. Trans. R. Soc. B Biol. Sci. 363, 557–572. 10.1098/rstb.2007.2170 PMC2610170 17715053

[pbi13170-bib-0008] Cooper, M. , Messina, C.D. , Podlich, D. , Totir, L.R. , Baumgarten, A. , Hausmann, N.J. , Wright, D. , et al. (2014) Predicting the future of plant breeding: complementing empirical evaluation with genetic prediction. Crop Pasture Sci. 65, 311–336.

[pbi13170-bib-0009] Cuevas, J. , Crossa, J. , Soberanis, V. , Pérez‐Elizalde, S. , Pérez‐Rodríguez, P. , Campos, G. , Montesinos‐López, O. , et al. (2016) Genomic prediction of genotype × environment interaction kernel regression models. Plant Genome, 9(3), 1–20. 10.3835/plantgenome2016.03.0024 27902799

[pbi13170-bib-0010] de los Campos, G. , Vazquez, A.I. , Fernando, R. , Klimentidis, Y.C. and Sorensen, D. (2013) Prediction of complex human traits using the genomic best linear unbiased predictor. PLoS Genet. 9, e1003608. 23874214 10.1371/journal.pgen.1003608 PMC3708840

[pbi13170-bib-0011] Duvick, D.N. (2001) Biotechnology in the 1930s: the development of hybrid maize. Nat. Rev. Genet. 2, 69–74. 11253074 10.1038/35047587

[pbi13170-bib-0012] Fujii, S. , Kazama, T. , Ito, Y. , Kojima, S. and Toriyama, K. (2014) A candidate factor that interacts with RF2, a restorer of fertility of Lead rice‐type cytoplasmic male sterility in rice. Rice, 7, 21–25. 26224552 10.1186/s12284-014-0021-6 PMC4884035

[pbi13170-bib-0013] Habier, D. , Fernando, R.L. and Garrick, D.J. (2013) Genomic BLUP decoded: a look into the black box of genomic prediction. Genetics, 194, 597–607. 23640517 10.1534/genetics.113.152207 PMC3697966

[pbi13170-bib-0014] Huang, X. , Yang, S. , Gong, J. , Zhao, Y. , Feng, Q. , Gong, H. , Li, W. , et al. (2015) Genomic analysis of hybrid rice varieties reveals numerous superior alleles that contribute to heterosis. Nat. Commun. 6, 6258–6268. 25651972 10.1038/ncomms7258 PMC4327311

[pbi13170-bib-0015] Kazama, T. and Toriyama, K. (2014) A fertility restorer gene, Rf4, widely used for hybrid rice breeding encodes a pentatricopeptide repeat protein. Rice, 7, 28–33. 26224557 10.1186/s12284-014-0028-z PMC4884050

[pbi13170-bib-0016] Khan, N.U. , Hassan, G. , Kumbhar, M.B. , Marwat, K.B. , Khan, M.A. , Parveen, A. and Saeed, M. (2009) Combining ability analysis to identify suitable parents for heterosis in seed cotton yield, its components and lint% in upland cotton. Ind. Crops Prod. 29, 108–115.

[pbi13170-bib-0017] Kohavi, R. (1995) A study of cross‐validation and bootstrap for accuracy estimation and model selection, pp. 1137–1145.

[pbi13170-bib-0018] Li, H. and Durbin, R. (2009) Fast and accurate short read alignment with Burrows‐Wheeler transform. Bioinformatics, 25, 1754–1760. 19451168 10.1093/bioinformatics/btp324 PMC2705234

[pbi13170-bib-0019] Li, Z.K. , Zhang, G.Y. and McNally, K.L. (2014) The 3,000 Rice Genomes project.

[pbi13170-bib-0020] Marulanda, J.J. , Mi, X. , Melchinger, A.E. , Xu, J. , Würschum, T. and Longin, C.F.H. (2016) Optimum breeding strategies using genomic selection for hybrid breeding in wheat, maize, rye, barley, rice and triticale. Theor. Appl. Genet. 129, 1901–1913. 27389871 10.1007/s00122-016-2748-5

[pbi13170-bib-0021] Massman, J.M. , Gordillo, A. , Lorenzana, R.E. and Bernardo, R. (2013) Genomewide predictions from maize single‐cross data. Theor. Appl. Genet. 126, 13–22. 22886355 10.1007/s00122-012-1955-y

[pbi13170-bib-0022] Pasanen, L. , Holmström, L. and Sillanpää, M.J. (2015) Bayesian LASSO, scale space and decision making in association genetics. PLoS ONE, 10, e0120017. 25856391 10.1371/journal.pone.0120017 PMC4391919

[pbi13170-bib-0023] SAS Institute Inc (2009). SAS/STAT: Users' Guide, Version 9.3. Cary, NC: SAS Institute Inc.

[pbi13170-bib-0024] Schrag, T.A. , Möhring, J. , Melchinger, A.E. , Kusterer, B. , Dhillon, B.S. , Piepho, H.‐P. and Frisch, M. (2010) Prediction of hybrid performance in maize using molecular markers and joint analyses of hybrids and parental inbreds. Theor. Appl. Genet. 120, 451–461. 19916002 10.1007/s00122-009-1208-x

[pbi13170-bib-0025] Speed, D. and Balding, D.J. (2015) Relatedness in the post‐genomic era: is it still useful? Nat. Rev. Genet. 16, 33–44. 25404112 10.1038/nrg3821

[pbi13170-bib-0026] Spindel, J. , Begum, H. , Akdemir, D. , Virk, P. , Collard, B. , Redoña, E. , Atlin, G. , et al. (2015) Genomic selection and association mapping in rice (Oryza sativa): effect of trait genetic architecture, training population composition, marker number and statistical model on accuracy of rice genomic selection in elite, tropical rice breeding lines. PLoS Genet. 11, e1004982. 25689273 10.1371/journal.pgen.1004982 PMC4334555

[pbi13170-bib-0027] Stephens, J. and Holland, R. (1954) Cytoplasmic male‐sterility for hybrid sorghum seed production. Agron. J. 46, 20–23.

[pbi13170-bib-0028] Stuber, C.W. (1994) Heterosis in plant breeding. Plant Breed Rev 12, 227–251.

[pbi13170-bib-0029] Sun, C. , Hu, Z. , Zheng, T. , Lu, K. , Zhao, Y. , Wang, W. , Shi, J. , et al. (2017) RPAN: rice pan‐genome browser for ∼3000 rice genomes. Nucleic Acids Res. 45, 597–605. 27940610 10.1093/nar/gkw958 PMC5314802

[pbi13170-bib-0030] Tu, J. , Zhang, G. , Datta, K. , Xu, C. , He, Y. , Zhang, Q. , Khush, G.S. , et al. (2000) Field performance of transgenic elite commercial hybrid rice expressing Bacillus thuringiensis δ‐endotoxin. Nat. Biotechnol. 18, 1101–1104. 11017051 10.1038/80310

[pbi13170-bib-0031] VanRaden, P.M. (2008) Efficient methods to compute genomic predictions. J. Dairy Sci. 91, 4414–4423. 18946147 10.3168/jds.2007-0980

[pbi13170-bib-0032] Vitezica, Z.G. , Varona, L. and Legarra, A. (2013) On the additive and dominant variance and covariance of individuals within the genomic selection scope. Genetics, 195, 1223–1230. 24121775 10.1534/genetics.113.155176 PMC3832268

[pbi13170-bib-0033] Wang, Z. , Zou, Y. , Li, X. , Zhang, Q. , Chen, L. , Wu, H. , Su, D. , et al. (2006) Cytoplasmic male sterility of rice with boro II cytoplasm is caused by a cytotoxic peptide and is restored by two related PPR motif genes via distinct modes of mRNA silencing. Plant Cell, 18, 676–687. 16489123 10.1105/tpc.105.038240 PMC1383642

[pbi13170-bib-0034] Wang, Y. , Mette, M.F. , Miedaner, T. , Gottwald, M. , Wilde, P. , Reif, J.C. and Zhao, Y. (2014) The accuracy of prediction of genomic selection in elite hybrid rye populations surpasses the accuracy of marker‐assisted selection and is equally augmented by multiple field evaluation locations and test years. BMC Genom. 15, 556. 10.1186/1471-2164-15-556 PMC4101178 24997166

[pbi13170-bib-0035] Wang, W. , Mauleon, R. , Hu, Z. , Chebotarov, D. , Tai, S. , Wu, Z. and Li, M. , et al. (2018) Genomic variation in 3,010 diverse accessions of Asian cultivated rice. Nature, 557, 43–49. 29695866 10.1038/s41586-018-0063-9 PMC6784863

[pbi13170-bib-0036] Wei, J. and Xu, S. (2016) A random‐model approach to QTL mapping in multiparent advanced generation intercross (MAGIC) populations. Genetics, 202, 471–486. 26715662 10.1534/genetics.115.179945 PMC4788229

[pbi13170-bib-0037] Xu, S. , Xu, Y. , Zhu, D. and Zhang, Q. (2014) Predicting hybrid performance in rice using genomic best linear unbiased prediction. Proc. Natl Acad. Sci. 111, 12456–12461. 25114224 10.1073/pnas.1413750111 PMC4151732

[pbi13170-bib-0038] Zhao, Y. , Li, Z. , Liu, G. , Jiang, Y. , Maurer, H.P. , Würschum, T. , Mock, H.‐P. , et al. (2015a) Genome‐based establishment of a high‐yielding heterotic pattern for hybrid wheat breeding. Proc. Natl Acad. Sci. 112, 15624–15629. 26663911 10.1073/pnas.1514547112 PMC4697414

[pbi13170-bib-0039] Zhao, Y. , Mette, M.F. and Reif, J.C. (2015b) Genomic selection in hybrid breeding. Plant Breed. 134, 1–10.

