# Supplementary material for: Hybrid breeding of rice via genomic selection
Source: Plant Biotechnol J. 2019 Jun 26;18(1):57–67. doi: 10.1111/pbi.13170 (PMC6920338; doi:10.1111/pbi.13170)
Supplement: Supplementary file 7 — Note S1 Additive & dominant (A‐D) model. Note S2 G×E interaction model. Table S1 Estimated parameters for 10 traits of the 1495 hybrids. Table S2 Prediction abilities of the test population (Pop 2) using phenotypes collected from different locations of the training population (Pop 1). Table S3 Correlation matrix between 10 agronomic traits. Table S4 Estimated parameters for 10 traits of the 1495 hybrids from the additive‐dominant (A‐D) model. Table S5 Information of 21 inbred lines of the half diallel cross experiment (Pop 2). Table S6 Information of 27 maintainer lines of the 3K rice genome project (Pop 3). Table S7 Distribution of SNPs on the 12 chromosomes of rice. Table S8 ANOVA table for estimating variance components that are required for estimating the broad sense heritability. Figure S1 Predicted phenotypes plotted against observed phenotypes of the 1495 hybrid rice for 10 traits (YD, PN, GN, SSR, KGW, HD, PH, PL, GL and GW). Figure S2 Prediction abilities using phenotypes collected from Hangzhou and Sanya separately and prediction abilities using mean phenotype of the two sites (pooled). Figure S3 Three‐line mating system. The three‐line mating system involves three lines. Figure S4 Distribution of the 3,000 rice accessions from five distinct groups of the 3K Rice Genome Project. Figure S5 Sketch of a K‐fold cross validation where K = 10. [file PBI-18-57-s006.docx]

**Hybrid Breeding of Rice via Genomic Selection**

Yanru Cui^1, 2*^, Ruidong Li^2*^, Guangwei Li^3*^, Fan Zhang^4^, Tiantian Zhu^2^, Qifa Zhang^3^, Jauhar Ali^5^, Zhikang Li^4, 6^ and Shizhong Xu^2^

^1^Hebei Agricultural University, Baoding 071001, China; ^2^Department of Botany and Plant Sciences, University of California, Riverside, CA 92521; ^3^National Key Laboratory of Crop Genetic Improvement and National Centre of Plant Gene Research (Wuhan), Huazhong Agricultural University, Wuhan 430070, China; ^4^Institute of Crop Science/National Key Facility for Crop Gene Resource and Genetic Improvement, Chinese Academy of Agricultural Sciences, Beijing 100081, China; ^5^International Rice Research Institute, DAPO Box 7777, Metro Manila, Philippines; and ^6^Anhui Agricultural University, Hefei 230036, China.

**Supporting Information**

**Supplementary Note S1: Additive & dominant (A-D) model**

The additive and dominance (A-D) model is defined as a model that includes both the additive and dominance effects while the model described in the main text contains only the additive effects. The linear mixed model for A-D is

$\begin{matrix} y= X\beta+\sum_{k=1}^{m} Z_{k}\gamma_{k}\text{+}\sum_{k=1}^{m} D_{k}\delta_{k}+\varepsilon\\ = X\beta+\xi+\zeta+\varepsilon\end{matrix}$ (S1)

where $y$ is an $n\times1$ vector of the phenotypic values for a quantitative trait measured from *n* individuals, *X* is a design matrix for all covariates, $\beta$ is a vector of the effects for the covariates (non-genetic effects, including the intercept), $Z_{k}=\left\{Z_{jk}\right\}$ is an $n\times1$ vector of additive genotype codes for marker *k* for individual *j*, $\gamma_{k}$ is the additive genetic effect of marker *k*, $D_{k}=\left\{D_{jk}\right\}$ is an $n\times1$ vector of dominant genotype codes, $\delta_{k}$ is the dominant effect of marker *k* and $\varepsilon$ is an $n\times1$ vector of residual errors with an assumed $N(0,I\sigma^{2})$ distribution. The additive numerical code for individual *j* at locus *k* is defined as $Z_{jk}=-1$ for $A_{1}A_{1}$, $Z_{jk}=0$ for $A_{1}A_{2}$, and $Z_{jk}=1$ for $A_{2}A_{2}$. Assume that the additive effects of all markers follow a normal distribution with zero mean and a common variance, , for all $k=1,2,...,m$, where is an unknown additive polygenic variance. Note that $\xi=\sum_{k=1}^{m} Z_{k}\gamma_{k}$ is an $n\times1$ vector of polygenic additive effects. The dominant numerical code for individual *j* at locus *k* is defined as $Z_{jk}=0$ for $A_{1}A_{1}$, $Z_{jk}=1$ for $A_{1}A_{2}$, and $Z_{jk}=0$ for $A_{2}A_{2}$. Assume that the dominant effects of all markers follow a normal distribution with zero mean and a common variance, $\delta_{k}\sim N(0,\tau^{2}/m)$, for all $k=1,2,...,m$, where $\tau^{2}$ is an unknown polygenic dominance variance. Note that $\zeta=\sum_{k=1}^{m} Z_{k}\delta_{k}$ is an $n\times1$ vector of polygenic dominant effects. The expectation of *y* is $\text{E}(y)=X\beta$ and the variance-covariance matrix of *y* is

(S2)

where

$K_{a}=\frac{1}{m}\sum_{k=1}^{m} Z_{k}Z_{k}^{T}$ and $K_{d}=\frac{1}{m}\sum_{k=1}^{m} D_{k}D_{k}^{T}$

are the additive and dominance kinship matrices, respectively. In addition, we also define and $\lambda_{d}=\tau^{2}/\sigma^{2}$ as variance ratios.

Let $y_{1}$ and $y_{2}$ be the phenotypic values of the training and test samples, respectively. All other matrices are partitioned accordingly. The A-D prediction model is

$\left[\begin{matrix} y_{1} \\ y_{2} \end{matrix}\right]=\left[\begin{matrix} X\beta_{1} \\ X\beta_{2} \end{matrix}\right]+\left[\begin{matrix} \xi_{1} \\ \xi_{2} \end{matrix}\right]+\left[\begin{matrix} \zeta_{1} \\ \zeta_{2} \end{matrix}\right]+\left[\begin{matrix} \varepsilon_{1} \\ \varepsilon_{2} \end{matrix}\right]$ (S3)

where

$E\left[\begin{matrix} y_{1} \\ y_{2} \end{matrix}\right]=\left[\begin{matrix} X\beta_{1} \\ X\beta_{2} \end{matrix}\right]$ (S4)

and

(S5)

where notation like $K_{a}^{12}$ indicates portioning of the corresponding kinship matrix according the training and test samples. The BLUP prediction of hybrids in the test population is expressed as

$\hat{y}_{2}=E\left(y_{2}\left|y_{1}\right.\right)=X_{2}\hat{\beta}+(\hat{\lambda}_{a}K_{a}^{21}+\hat{\lambda}_{d}K_{d}^{21})(K_{a}^{11}\hat{\lambda}_{a}+K_{d}^{11}\hat{\lambda}_{d}+I)^{-1}(y_{1}-X_{1}\hat{\beta})$ (S6)

**Supplementary Note S2: G×E interaction model**

The G×E interaction model was directly adopted from Cuevas et al. (2016) using a compact form of matrix notation. The hybrid rice experiment was replicated in two locations and thus we have two environments. Let $y_{1}$ and $y_{2}$ be the $n\times1$ vectors of phenotypic values of all *n* hybrids in the two environments, respectively. Before introducing the G×E model, let us review the additive model in one environment using matrix notation, say the *r*th environment,

$$y_{r}=X\beta_{r}+Z\gamma_{r}+\varepsilon_{r}$$

for $r=1,2$, where $y_{r}$ is an $n\times1$ vector (not a single value) of phenotypic values of all *n* hybrids collected from the *r*th environment, $\beta_{r}$ is a vector of environment specific effects (fixed), $Z$ is an $n\times m$ matrix of genotype indicator variables (additive code only), $\gamma_{r}$ is an $m\times1$ vector of marker effects in the *r*th environment and $\varepsilon_{r}$ is an $n\times1$ vector of residual errors. After incorporating the two environments together, the G×E interaction model becomes

$$\left[\begin{matrix} y_{1} \\ y_{2} \end{matrix}\right]=\left[\begin{matrix} X & 0 \\ 0 & X \end{matrix}\right]\left[\begin{matrix} \beta_{1} \\ \beta_{2} \end{matrix}\right]+\left[\begin{matrix} Z \\ Z \end{matrix}\right]\alpha+\left[\begin{matrix} Z & 0 \\ 0 & Z \end{matrix}\right]\left[\begin{matrix} \gamma_{1} \\ \gamma_{2} \end{matrix}\right]+\left[\begin{matrix} 1_{n} & 0 \\ 0 & 1_{n} \end{matrix}\right]\left[\begin{matrix} \varepsilon_{1} \\ \varepsilon_{2} \end{matrix}\right]$$

Obviously, there is an additional term $\alpha$ (an $m\times1$ vector) representing the main effects of all markers. The corresponding environment specific effects are represented by $\gamma_{r}$ for $r=1,2$. When a marker has different effects in different environments, G×E interaction occurs, per definition of G×E interaction. Let and for $r=1,2$, where is the main effect polygenic variance and is the environment specific polygenic variance. In addition, $\varepsilon_{r}\sim N(0,I\sigma_{r}^{2})$, where $\sigma_{r}^{2}$ is an environment specific residual error variance. In general, there are covariance terms for the environment specific polygenes and residual errors represented by and $\sigma_{12}^{2}$, respectively. They were assumed to be zero in this study, following the GBLUP model of Cuevas et al. (2016). The expectation of the above model is

$$\text{E}\left[\begin{matrix} y_{1} \\ y_{2} \end{matrix}\right]=\left[\begin{matrix} X & 0 \\ 0 & X \end{matrix}\right]\left[\begin{matrix} \beta_{1} \\ \beta_{2} \end{matrix}\right]$$

The variance-covariance matrix of the model is

where represents Kronecker matrix multiplication and

, and $\Sigma=\left[\begin{matrix} \sigma_{1}^{2} & \sigma_{12}^{2} \\ \sigma_{21}^{2} & \sigma_{2}^{2} \end{matrix}\right]$

The kinship matrix remains the same as defined before $K=m^{-1}\sum_{k=1}^{m} Z_{k}Z_{k}^{T}$. Here, we assumed (Cuevas et al. 2016). To implement the G×E interaction model using existing software packages, the variance-covariance matrix is expressed as a linear combination of all variance components, as shown below,

Once parameters were estimated from the restricted maximum likelihood method (REML) method, they were used for BLUP prediction of a test sample from a training sample via the conditional expectation of multivariate normal distribution as described in the main text. From the G×E model, we obtained $\hat{y}_{1}$ and $\hat{y}_{2}$ drawn from 10-fold cross validations. Predictability for each environment was calculated from the correlation coefficient between the predicted and the observed phenotypic values within each environment. These prediction abilities were then compared with the prediction ability of each environment evaluated alone with environment specific data.

**Supplementary Table S1** Estimated parameters for 10 traits of the 1495 hybrids

| Trait |  |  | $\sigma^{2}$ | $\lambda$ |
| --- | --- | --- | --- | --- |
| Yield | 35.900 | 194.000 | 57.800 | 3.350 |
| PN | 13.800 | 33.500 | 3.870 | 8.640 |
| GN | 124.000 | 8,380.000 | 218.000 | 38.400 |
| SSR | 0.787 | 0.022 | 0.003 | 7.040 |
| TGW | 27.100 | 88.700 | 0.571 | 155.000 |
| HD | 73.400 | 723.000 | 2.950 | 245.000 |
| PH | 91.400 | 936.000 | 8.070 | 116.000 |
| PL | 20.300 | 26.700 | 0.714 | 37.400 |
| GL | 7.300 | 2.780 | 0.021 | 130.000 |
| GW | 3.160 | 0.266 | 0.001 | 240.000 |

Note: The estimated values of * , $\sigma^{2}$  *and *λ*  will be different by using different genotype coding systems, but the prediction ability will not be affected by the coding system.

**Supplementary Table S2** Prediction abilities of the validation population (Pop 2) using phenotypes collected from different locations of the training population (Pop 1)

| Location | Yield | GN | TGW | PH | GL | GW |
| --- | --- | --- | --- | --- | --- | --- |
| Pooled | 0.542 | 0.616 | 0.544 | 0.580 | 0.916 | 0.866 |
| Sanya | 0.485 | 0.561 | 0.482 | 0.480 | 0.913 | 0.860 |
| Hangzhou | 0.554 | 0.316 | 0.566 | 0.637 | 0.913 | 0.863 |

**Supplementary Table S3** Correlation matrix between 10 agronomic traits

| Trait | Yield | PN | GN | SSR | TGW | HD | PH | PL | GL | GW |
| --- | --- | --- | --- | --- | --- | --- | --- | --- | --- | --- |
| Yield | 1 | 0.518 | 0.346 | 0.244 | 0.173 | 0.240 | 0.389 | 0.286 | 0.043 | 0.104 |
| PN | 0.518 | 1 | -0.223 | -0.179 | -0.229 | 0.001 | -0.126 | -0.113 | -0.091 | -0.173 |
| GN | 0.346 | -0.223 | 1 | -0.164 | -0.218 | 0.220 | 0.269 | 0.184 | -0.151 | -0.062 |
| SSR | 0.244 | -0.179 | -0.164 | 1 | 0.116 | 0.052 | 0.113 | 0.043 | -0.057 | 0.131 |
| TGW | 0.173 | -0.229 | -0.218 | 0.116 | 1 | 0.111 | 0.509 | 0.446 | 0.547 | 0.497 |
| HD | 0.240 | 0.001 | 0.220 | 0.052 | 0.111 | 1 | 0.445 | 0.191 | -0.093 | 0.213 |
| PH | 0.389 | -0.126 | 0.269 | 0.113 | 0.509 | 0.445 | 1 | 0.608 | 0.219 | 0.320 |
| PL | 0.286 | -0.113 | 0.184 | 0.043 | 0.446 | 0.191 | 0.608 | 1 | 0.424 | 0.092 |
| GL | 0.043 | -0.091 | -0.151 | -0.057 | 0.547 | -0.093 | 0.219 | 0.424 | 1 | -0.398 |
| GW | 0.104 | -0.173 | -0.062 | 0.131 | 0.497 | 0.213 | 0.320 | 0.092 | -0.398 | 1 |

Note: The correlation matrix is symmetrical, i.e., the correlation between *x* and *y* is the same as the correlation between *y* and *x*.

**Supplementary Table S4** Estimated parameters for 10 traits of the 1495 hybrids from the additive-dominant (A-D) model

| Trait | $\beta$ | $\sigma_{A}^{2}$ | $\sigma_{D}^{2}$ | $\sigma_{E}^{2}$ |
| --- | --- | --- | --- | --- |
| Yield | 34.2000 | 119.0000 | 14.8000 | 54.7000 |
| PN | 13.3000 | 28.1000 | 0.8430 | 3.7400 |
| GN | 123.0000 | 5,810.0000 | 244.0000 | 197.0000 |
| SSR | 0.8030 | 0.0133 | 0.0013 | 0.0031 |
| TGW | 27.1000 | 74.4000 | 1.2400 | 0.4590 |
| HD | 76.2000 | 521.0000 | 21.2000 | 0.9400 |
| PH | 91.1000 | 673.0000 | 19.8000 | 6.9200 |
| PL | 20.4000 | 24.6000 | 0.2360 | 0.6860 |
| GL | 7.4100 | 2.3300 | 0.0380 | 0.0180 |
| GW | 3.1300 | 0.2130 | 0.0014 | 0.0014 |

$\beta$ – intercept; $\sigma_{A}^{2}$ – additive variance; $\sigma_{D}^{2}$ – dominance variance; $\sigma_{E}^{2}$ – residual error variance

**Supplementary Table S5** Information of 21 inbred lines of the half diallel cross experiment (Pop 2)

| Code | Variety | Subspecies | Description | Sourrce |
| --- | --- | --- | --- | --- |
| LGW1 | Zhenshan 97 | *Indica* | Cultivar | China |
| LGW2 | Minghui 63 | *Indica* | Cultivar | China |
| LGW3 | 9311 | *Indica* | Cultivar | China |
| LGW4 | Nanjing 11 | *Indica* | Cultivar | China |
| LGW5 | IR64 | *Indica* | Cultivar | Philippines |
| LGW6 | Teqing | *Indica* | Cultivar | China |
| LGW7 | Swarna | *Indica* | Cultivar | Philippines |
| LGW8 | Varylava | *Japonica* | Cultivar | Malaysia |
| LGW9 | Huanghuazhan | *Indica* | Cultivar | China |
| LGW10 | Kasalath | *Indica* | Landrace | India |
| LGW11 | Guangluai 4 | *Indica* | Cultivar | China |
| LGW12 | Balilla | *Japonica* | Cultivar | Italy |
| LGW13 | Niponbare | *Japonica* | Cultivar | Japan |
| LGW14 | Zhonghua 11 | *Japonica* | Cultivar | China |
| LGW15 | Wuyungeng 7 | *Japonica* | Cultivar | China |
| LGW16 | Kongyu 131 | *Japonica* | Cultivar | China |
| LGW17 | Koshihikari | *Japonica* | Cultivar | Japan |
| LGW18 | Taichung 65 | *Japonica* | Cultivar | China |
| LGW19 | 02428 | *Japonica* | Cultivar | China |
| LGW20 | Dular | *Indica* | Landrace | India |
| LGW21 | Lemont | *Japonica* | Cultivar | America |

**Supplementary Table S6.** Information of 27 maintainer lines of the 3K rice genome project (Pop 3)

| ID | Name | Subspecies | Origin |
| --- | --- | --- | --- |
| B137 | 80B | *Indica* | Phillipines |
| B150 | L301B | *Indica* | China |
| B151 | Jin Nante43B | *Indica* | China |
| B153 | Qing Siai16B | *Indica* | China |
| B154 | LingmingB | *Temperate japonica* | China |
| B155 | Baoxie-7B | *Indica* | China |
| B156 | G-Zhan Shan97B | *Indica* | China |
| B157 | 88B | *Indica* | China |
| B179 | KeluoduoB | *Temperate japonica* | France |
| B180 | P1790-5-1M-4-5M-1B-3M-B | *Indica* | Columbia |
| B195 | PMS 10B | *Indica* | India |
| B202 | Baoxie123B | *Indica* | China |
| B247 | Jin NanteB | *Indica* | China |
| B248 | ZhuzhenB | *Indica* | China |
| B249 | Chaoyang1B | *Indica* | China |
| B250 | Shennong WangengB | *Temperate japonica* | China |
| B252 | XiangaiB | *Indica* | China |
| B253 | Jiangnong zao1B | *Indica* | China |
| B254 | JinghuB | *Indica* | China |
| B255 | Dianrui409B | *Indica* | China |
| CX10 | Gang46B | *Indica* | China |
| CX43 | IR66897B | *Indica* | Phillipines |
| CX44 | IR58025B | *Indica* | Phillipines |
| CX133 | Zhanshan97B | *Indica* | China |
| CX281 | IR68897B | *Indica* | Phillipines |
| CX124 | IR71466-75-3-B-1 | *Indica* | Phillipines |
| CX242 | IR47686-4-4-B-1 | *Intermediate type* | Phillipines |

**Supplementary Table S7** Distribution of SNPs on 12 chromosomes of rice

| Chromosome | Number of SNPs |
| --- | --- |
| 1 | 24566 |
| 2 | 22655 |
| 3 | 23062 |
| 4 | 16663 |
| 5 | 17328 |
| 6 | 17065 |
| 7 | 14785 |
| 8 | 15226 |
| 9 | 11970 |
| 10 | 11211 |
| 11 | 14068 |
| 12 | 13157 |
| Total | 201756 |

**Supplementary Table S8** ANOVA table for estimating variance components that are required in obtaining the broad sense heritability.

| Source | Df | SS | MS | E(MS)^a^ |
| --- | --- | --- | --- | --- |
| Between hybrids | 1495-1 | SS_G_ | MS_G_ | $\sigma_{E}^{2}+2\sigma_{G}^{2}$ |
| Between blocks | 2-1 | SS_B_ | MS_B_ |  |
| Residual | (1495-1)(2-1) | SS_E_ | MS_E_ | $\sigma_{E}^{2}$ |

^a^ The estimated variances are obtained via $\hat{\sigma}_{E}^{2}=MS_{E}$ and $\hat{\sigma}_{G}^{2}=(MS_{G}-MS_{E})/2$.

Df: degree of freedom; SS: sum of squares; MS: mean squares; E(MS): expected mean squares.


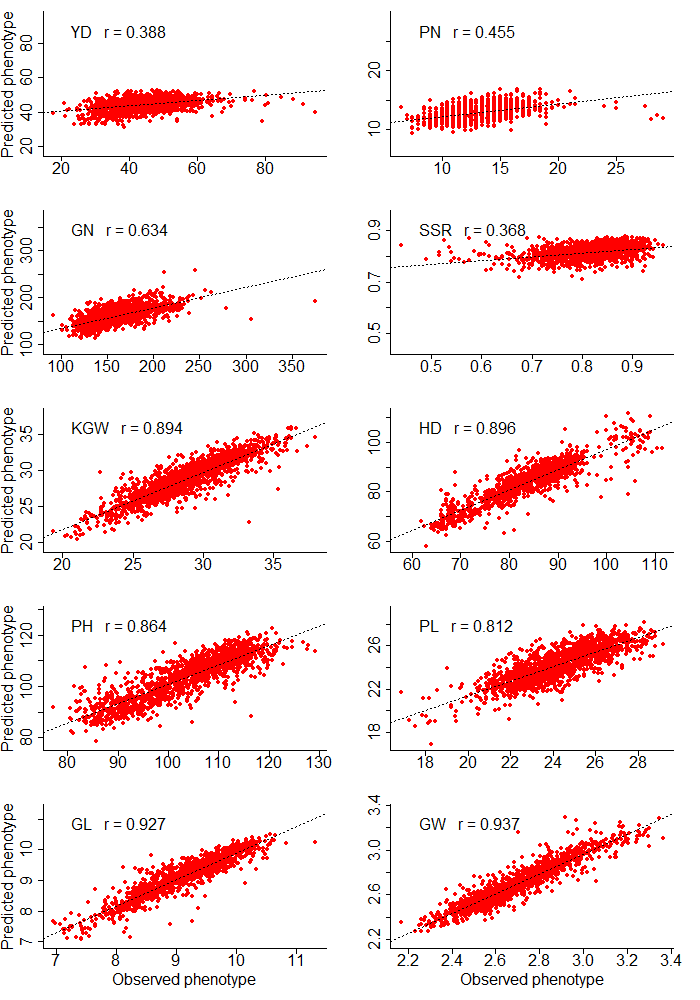


**Supplementary Fig. S1** Predicted phenotypes plotted against observed phenotypes of the 1495 hybrid rice for 10 traits (YD, PN, GN, SSR, KGW, HD, PH, PL, GL and GW).


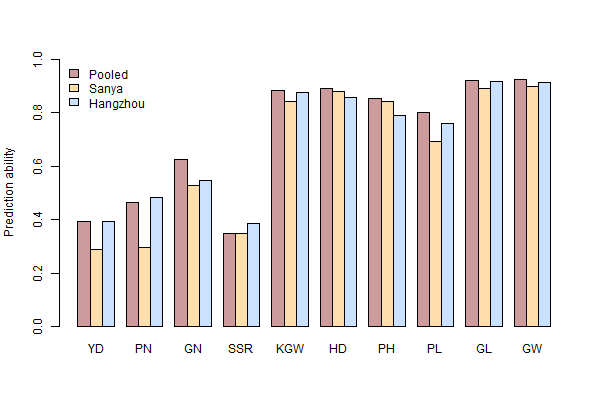


**Supplementary Fig. S2** Prediction abilities using phenotypes collected from Hangzhou and Sanya separately and prediction abilities using mean phenotype of the two sites (pooled)


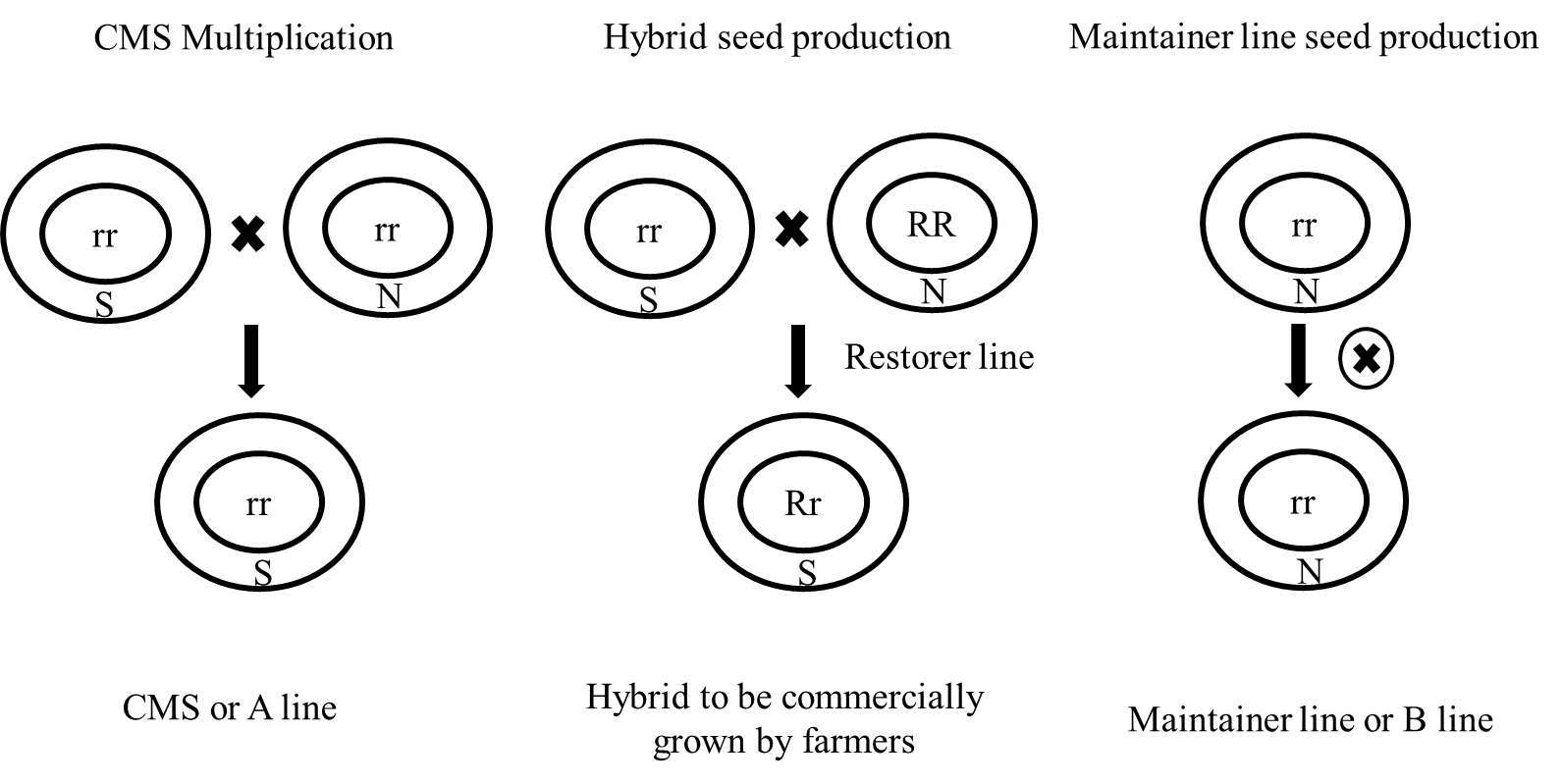


**Supplementary Fig. S3** Three-line mating system

The three-line mating system involves three lines. The cytoplasmic male sterility (CMS) is the result of interaction between specific sterility inducing cytoplasm gene (S) and the recessive nuclear gene (rr). The maintainer lines have the same unclear gene as the CMS lines and thus can reserve the sterility of CMS. The restorer lines possess the restorer gene (RR) which can be utilized as the male parent used to cross with CMS to produce hybrids.


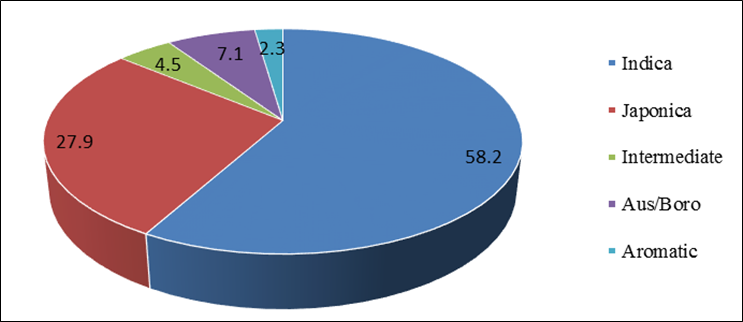


**Supplementary Fig. S4** Distribution of the 3,000 rice accessions from five distinct groups of the 3K Rice Genome Project


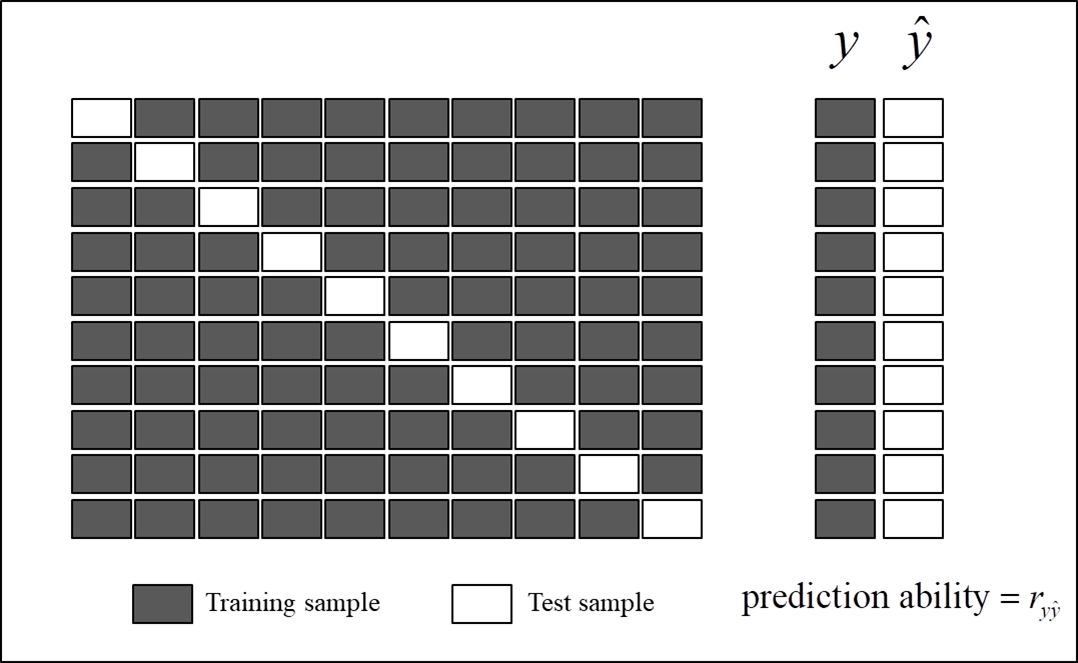


**Supplementary Fig. S5** Sketch of a K-fold cross validation where K = 10.

In a 10-fold cross validation, the population is partitioned into 10 parts. Using 9 parts to predict the remaining part. The process continues in turn until all parts are predicted. The prediction ability is defined as the correlation between the observed trait values and the predicted trait values.
